# An examination of prostate cancer trends in Australia, England, Canada and USA: Is the Australian death rate too high?

**DOI:** 10.1007/s00345-015-1514-7

**Published:** 2015-02-20

**Authors:** E. Feletto, A. Bang, D. Cole-Clark, V. Chalasani, K. Rasiah, D. P. Smith

**Affiliations:** 1 Cancer Research Division, Cancer Council NSW, Woolloomooloo, NSW Australia; 2 Department of Surgery, Royal North Shore Hospital, St Leonards, NSW Australia; 3 Australian and New Zealand Urogenital and Prostate (ANZUP) Cancer Trials Group, Discipline of Surgery, University of Sydney, Camperdown, NSW Australia; 4 Northern Sydney Local Health District, St Leonards, NSW Australia; 5 Kinghorn Cancer Centre, Garvan Institute of Medical Research, St Leonards, NSW Australia; 6 Griffith Health Institute, Griffith University, Nathan, QLD Australia

**Keywords:** Prostatic neoplasms, Mortality, Australia, USA, England, Canada

## Abstract

**Purpose:**

To compare prostate cancer incidence and mortality rates in Australia, USA, Canada and England and quantify the gap between observed prostate cancer deaths in Australia and expected deaths, using US mortality rates.

**Methods:**

Analysis of age-standardised prostate cancer incidence and mortality rates, using routinely available data, in four similarly developed countries and joinpoint regression to quantify the changing rates (annual percentage change: APC) and test statistical significance. Expected prostate cancer deaths, using US mortality rates, were calculated and compared with observed deaths in Australia (1994–2010).

**Results:**

In all four countries, incidence rates initially peaked between 1992 and 1994, but a second, higher peak occurred in Australia in 2009 (188.9/100,000), rising at a rate of 5.8 % (1998–2008). Mortality rates in the USA (APC: −2.9 %; 2004–2010), Canada (APC: −2.9 %; 2006–2011) and England (APC: −2.6 %; 2003–2008) decreased at a faster rate compared with Australia (APC: −1.7 %; 1997–2011). In 2010, mortality rates were highest in England and Australia (23.8/100,000 in both countries). The mortality gap between Australia and USA grew from 1994 to 2010, with a total of 10,895 excess prostate cancer deaths in Australia compared with US rates over 17 preceding years.

**Conclusions:**

Prostate cancer incidence rates are likely heavily influenced by prostate-specific antigen testing, but the fall in mortality occurred too soon to be solely a result of testing. Greater emphasis should be placed on addressing system-wide differences in the management of prostate cancer to reduce the number of men dying from this disease.

## Purpose

Over the past two decades, prostate cancer incidence rates have risen substantially in many developed countries. However, trends in mortality have been less dramatic and show considerable variation, especially between developed countries [1]. In Australia, prostate cancer incidence rose 144 % from 1982 to 2009, while mortality decreased 30 % from 1993 to 2010 [2]. Australian prostate cancer incidence rates are among the highest internationally, yet the reduction in mortality appears somewhat modest compared with other developed countries. Differences in prostate-specific antigen (PSA) testing practices appear to account for variations in incidence, whereas differences in mortality are not so clearly explained [3]. It has been proposed that differential mortality could be partially attributed to earlier detection and improvements in surgical and radiotherapy methods used for localised prostate cancer and increased the use of androgen deprivation therapy (ADT) and chemotherapy for men with later stage disease [4–6].

To quantify the apparent disparity between Australia and other developed countries in prostate cancer mortality, we analysed and compared incidence and mortality data in four countries. All four countries were recently classified as experiencing “earlier mortality decline” [1] for prostate cancer. Our aims were to:

1. Describe the trends in incidence and mortality rates from prostate cancer in four developed countries—Australia, England, USA and Canada—to better understand the differences in mortality between Australia and these countries;
2. Quantify the gap between observed prostate cancer deaths in Australia and expected deaths, using the country with the largest fall in mortality rates (USA) as a benchmark.

## Methods

In addition to Australia, the three nations were selected from the larger group 15 countries with “earlier mortality decline” as they have similar demographic compositions but had differing approaches to PSA testing from the 1990s compared to Australia: USA had high screening rates associated with higher awareness and media attention given to PSA testing, Canada also had high screening rates but with regional variation [7], and England had notably lower rates of PSA testing [8, 9]. Data on the number of prostate cancer deaths and newly diagnosed cases by 5-year age group and year were obtained for all four countries from publically available sources (Table 1). The age-standardised rates for each country were calculated using the European population as the reference population [10]. We also subdivided the data into two age groups for more detailed analysis—under 65 and over 65 years for incidence and under 75 and over 75 years for mortality. Subgroupings were based on the median ages of diagnosis and death for Australian men.

**Table 1:** Data sources

	Australia	England	Canada	USA
Cancer incidence				
Source	Australian Institute of Health and Welfare (AIHW)	Office of National Statistics (ONS)	Statistics Canada’s Canadian Socio-economic Information Management System (CANSIM)	Surveillance, Epidemiology and End Results (SEER) Program’s SEER*Stat
Years	1982–2010	1987–2011	1969–2007	1973–2010
Cancer mortality				
Source	AIHW	ONS	CANSIM	SEER*Stat
Years	1969–2011	1987–2010	1974–2011	1969–2010
Population figures	AIHW	ONS	Statistics Canada	SEER*Stat

Joinpoint regression was used to quantify the gradient of change in incidence and mortality rates over time and test their statistical significance. Joinpoint regression summarised trends over successive segments of time, and the annual percentage change (APC) was calculated for each time segment. The joinpoint regression was not restricted and allowed for the best fit, that is, where the APC was significantly different from 0 at *α* = 0.05 [11].

In addition, the number of observed prostate cancer deaths in Australia from 1994 to 2010 was compared with the number of expected deaths, using age-specific mortality rates from the USA and the Australian population by year. A similar method has previously been used by Sitas et al. [12] to quantify the change in Australian cancer incidence and mortality over 20 years. The year 1994 was chosen as it marked the first year of decline after the peak of prostate cancer mortality in Australia. The USA was selected as the benchmark as their mortality rates were the lowest of the four countries used in this study. This allowed us to quantify the gap between observed deaths in Australia and the number of deaths expected if Australia had experienced the same age-specific rates by year as in the USA. The standardised mortality ratio was calculated to test for statistical significance, with 95 % confidence intervals. No ethical approval was required for this study.

## Results

**Fig. 1:**
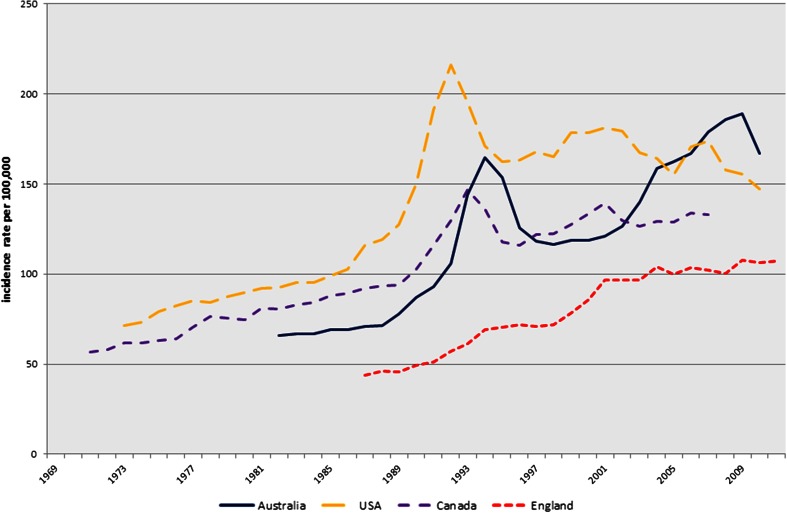
Age-standardised prostate cancer incidence rates

The age-standardised incidence rates and joinpoint regression for incidence in all four countries using all available data are shown in Fig. 1 and Table 2, respectively. Incidence in the USA initially peaked earlier (1992: 216.2/100,000) than Australia (1994: 164.9/100,000) and Canada (1993: 146.7/100,000), whereas England had a steady incline from 1987 onwards. From 1990–1994 to 1994–1998, the gradient of change in Australian incidence rate in men of all ages dropped from 20.1 %, largely attributed to a sharp rise in men under 65 years of age (APC: 41.9 %), to a more modest decline in men of all ages (APC: −10.1 %). However, the incidence did not return to the levels reported prior to the 1994 peak. Both Canada and the USA had periods of falling incidence in the early 1990s and again in the 2000s. Only the USA has had a continuing reduction in incidence in the most recent period (APC: −1.6 %; 2000–2010). Canadian rates were decreasing, and recent figures showed increasing incidence in men under 65 (APC: 2.0 %; 2001–2007). In contrast to the sharp peak seen in the other three countries, the incidence rate in England had a steadier incline and continued to rise, albeit at a lower rate of change (APC: 1.1 %; 2001–2011). The most recent available incidence data showed that the age-standardised rates were highest in Australia at 167/100,000 (2010), followed by USA at 147.2/100,000 (2010) then Canada at 133/100,000 (2007) and lowest in England at 107.4/100,000 (2011).

**Table 2:** Australia, England, Canada and US prostate cancer incidence trends (joinpoint regression): all years available

	Joinpoint 1	APC (95 % CI)	Joinpoint 2	APC (95 % CI)	Joinpoint 3	APC (95 % CI)	Joinpoint 4	APC (95 % CI)	Joinpoint 5	APC (95 % CI)	Joinpoint 6	APC (95 % CI)
Under 65												
Australia	1982–1991	4.5 (1.8,7.3)*	1991–1994	41.9 (16.1,73.5)*	1994–1999	−3.8 (−8.3,0.9)	1999–2008	10.3 (8.9,11.7)*	2008–2010	−2.2 (−10.2,6.4)		
England	1987–2004	10.0 (9.3,10.8)*	2004–2011	2.7 (1.3,4.3)*								
Canada	1971–1990	3.4 (3.0,3.8)*	1990–1993	25.4 (15.3,36.4)*	1993–1996	−2.7 (−9.4,4.5)	1996–2001	7.8 (5.7,10.0)*	2001−2007	2.0 (1.2,2.9)*		
USA	1973–1988	3.3 (2.3,4.3)*	1988–1992	22.9 (13.6,33.0)*	1992–2001	3.3 (2.0,4.6)*	2001–2010	−0.5 (−1.3,0.3)				
Over 65												
Australia	1982–1989	1.5 (−0.9,4.0)	1989–1994	15.5 (10.9,20.2)*	1994–1997	−13.1 (−22.3,–2.8)*	1997–2001	0.5 (−5.2,6.6)	2001–2008	4.4 (2.6,6.2)*	2008–2010	−8.1 (−16.3,0.9)
England	1987–1991	4.0 (0.8,7.3)*	1991–1994	10.3 (1.3,20.0)*	1994–1998	−0.1 (−3.9,3.8)	1998–2001	7.9 (0.5,15.9)*	2001–2011	0.1 (−0.4,0.6)		
Canada	1971–1990	2.9 (2.6,3.2)*	1990–1993	10.4 (3.8,17.4)*	1993–1996	−9.8 (−15.1,−4.2)*	1996–2000	2.7 (−0.3,5.8)	2000–2003	−4.0 (−9.1,1.4)	2003–2007	−0.0 (−1.7,1.7)
USA	1973–1988	2.8 (2.2,3.4)*	1988–1992	16.3 (10.7,22.1)*	1992–1995	−13.9 (−21.4,−5.7)*	1995–2000	1.5 (−1.6,4.6)	2000–2010	−2.5 (−3.2,−1.7)*		
All Ages												
Australia	1982–1990	2.8 (0.7,4.9)*	1990–1994	20.1 (12.5,28.3)*	1994–1998	−10.1 (−15.1,−4.8)*	1998–2008	5.8 (4.8,6.8)*	2008–2010	−4.8 (−12.4,3.5)		
England	1987–1991	3.9 (0.3,7.6)*	1991–1994	11.0 (0.9,22.1)*	1994–1998	0.7 (−3.5,5.1)	1998–2001	10.3 (2.0,19.3)*	2001–2011	1.1 (0.6,1.7)*		
Canada	1971–1990	3.0 (2.7,3.3)*	1990–1993	13.1 (6.6,20.0)*	1993–1996	−7.8 (−12.8,−2.5)*	1996–2001	3.6 (1.9,5.4)*	2001–2004	−2.5 (−7.2,2.4)	2004–2007	1.9 (−0.4,4.3)
USA	1973–1988	2.9 (2.2,3.6)*	1988–1992	17.9 (11.6,24.6)*	1992–1995	−9.9 (−18.4,−0.5)*	1995–2000	2.7 (−0.6,6.0)	2000–2010	−1.6 (−2.4,−0.9)*		

* Denotes a statistically significant result

**Fig. 2:**
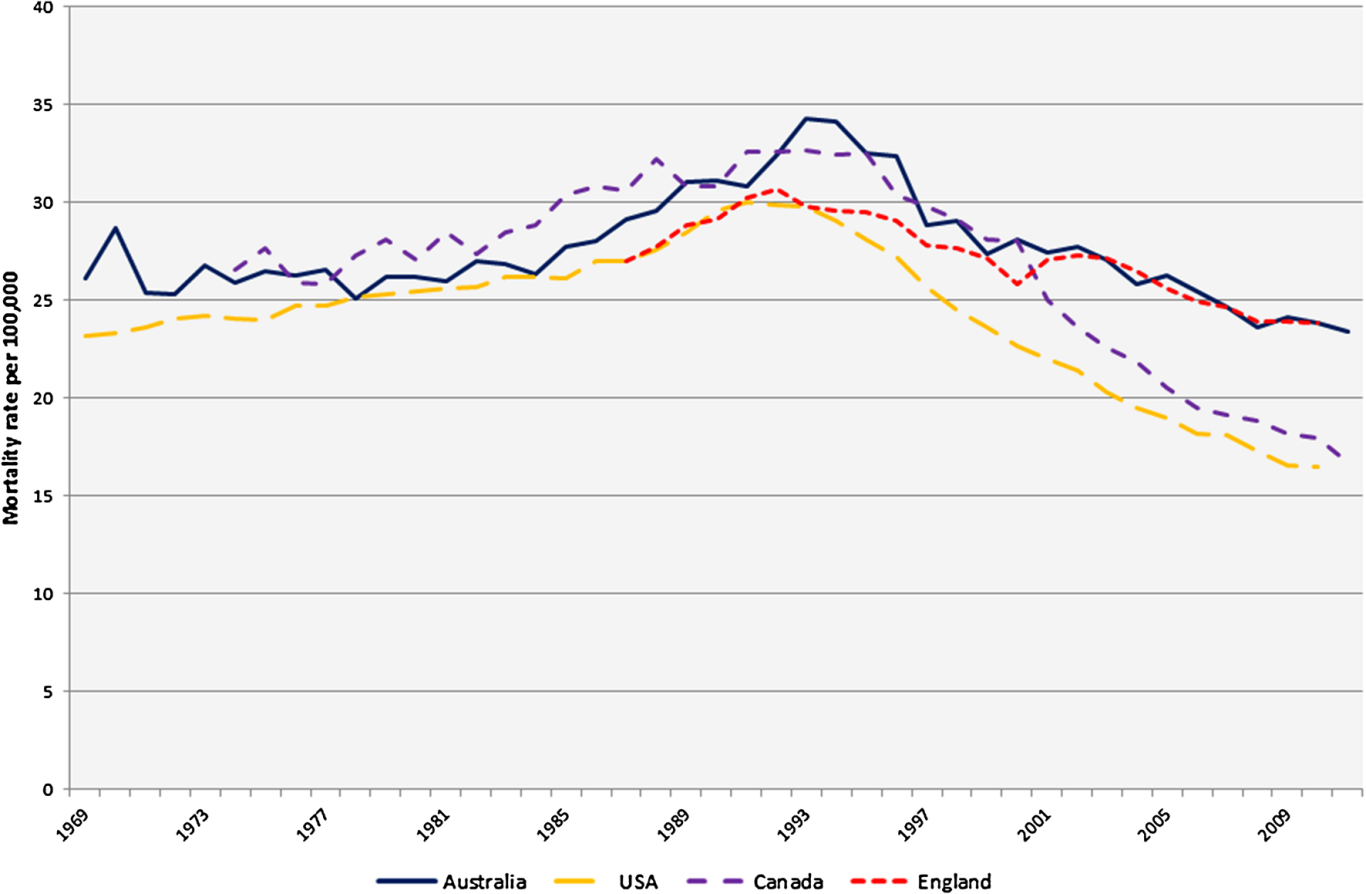
Age-standardised prostate cancer mortality rates

The standardised mortality rates and joinpoint regression for mortality in all four countries using all available data are shown in Fig. 2 and Table 3, respectively. Mortality peaked in the early 1990s for all four countries, firstly in the USA (1991: 30/100,000), then England (1992: 30.7/100,000) with Australia (34.3/100,000) and Canada (32.6/100,000) both reaching the highest mortality rate in 1993. The most recent available data showed that the age-standardised mortality rates were highest in England at 23.8/100,000 (2010), with Australia following closely behind at 23.4/100,000 (2011) and lowest in the USA at 16.5/100,000 (2010), with a similar low rate in Canada at 16.7/100,000 (2011).

**Table 3:** Australia, England, Canada and US prostate cancer mortality trends (joinpoint regression): all years available

	Joinpoint 1	APC (95 % CI)	Joinpoint 2	APC (95 % CI)	Joinpoint 3	APC (95 % CI)	Joinpoint 4	APC (95 % CI)	Joinpoint 5	APC (95 % CI)	Joinpoint 6	APC (95 % CI)
Under 75												
Australia	1969–1983	−0.3 (−0.9,0.5)	1983–1992	3.0 (1.6,4.4)*	1992–2011	−2.9 (−3.2,−2.6)*						
England	1987–1991	2.3 (0.5,4.2)*	1991–2010	−1.9 (−2.1,−1.8)*								
Canada	1974–1983	0.2 (−0.7,1.1)	1983–1986	5.8 (−3.0,15.4)	1986–1994	−0.4 (−1.4,0.7)	1994–2011	−4.2 (−4.5,−3.9)*				
USA	1969–1984	0.3 (0.2,0.5)*	1984–1991	1.7 (1.1,2.2)*	1991–1996	−3.5 (−4.4,−2.5)*	1996–2000	−6.0 (−7.6,−4.4)*	2000–2004	−3.7 (−5.4,−1.9)*	2004–2010	−2.0 (−2.7,−1.4)*
Over 75												
Australia	1969–1984	0.1 (−0.4,0.7)	1984–1994	2.6 (1.7,3.6)*	1994–1998	−4.0 (−7.9,0.1)	1998–2011	−1.1 (−1.4,−0.7)*				
England	1987–1992	3.2 (2.2,4.3)*	1992–2000	−1.5 (−2.1,−1.0)*	2000–2003	1.6 (−2.2,5.5)	2003–2008	−2.6 (−3.8,−1.5)*	2008–2010	1.1 (−2.4,4.8)		
Canada	1974–1995	1.3 (1.0,1.6)*	1995–2006	−4.4 (−5.1,−3.8)*	2006–2011	−2.8 (−4.5,−1.1)*						
USA	1969–1987	1.1 (1.0,1.2)*	1987–1993	2.8 (2.1,3.4)*	1993–2010	−3.5 (−3.6,−3.4)*						
All Ages												
Australia	1969–1983	−0.0 (−0.5,0.4)	1983–1994	2.5 (2.0,3.0)*	1994–1997	−5.1 (−10.3,0.4)	1997–2011	−1.7 (−1.9,−1.4)*				
England	1987–1992	2.6 (1.8,3.3)*	1992–2000	−1.9 (−2.3,−1.5)*	2000–2003	0.9 (−2.1,3.9)	2003–2008	−2.6 (−3.5,−1.7)*	2008–2010	0.0 (−2.8,3.0)		
Canada	1974–1995	1.2 (0.9,1.4)*	1995–2006	−4.6 (−5.1,−4.1)*	2006–2011	−2.9 (−4.4,−1.4)*						
USA	1969–1987	0.8 (0.7,0.9)*	1987–1991	2.9 (1.7,4.2)*	1991–1994	−1.2 (−3.4,1.1)	1994–2004	−4.0 (−4.2,−3.7)*	2004–2010	−2.9 (−3.3,−2.5)*		

* Denotes a statistically significant result

In the most recent time period, the rate of change in Australian mortality slowed (APC: −1.7 %; 1997–2011), as did American mortality which dropped from −4.0 % in 1994–2004 to −2.9 % in 2004–2010. In Canada there tended to be a slight increase on a similar trajectory compared to that seen prior to the highest mortality rate reported in 1993, whereas the consistent mortality reduction in England was interrupted by a period of increase from 2000 to 2003 (not statistically significant).

To quantify the absolute difference in the number of deaths between the USA and Australia, we obtained the number of observed deaths from prostate cancer in Australia from 1994 to 2010 and the number of expected deaths, after applying the annual US age-specific mortality rates. There were an additional 10,895 deaths in Australian men than would have been expected during the 17 years of this study. The difference in number of deaths rose from 393 deaths (17.7 %) in 1994 to 1,042 deaths (47.8 %) in 2010.

## Discussion

Our analysis showed that while mortality rates in Australia have decreased from 1994 onwards, they have done so at a different trajectory to rates in the USA, Canada or England. The US mortality rate (per 100,000) dropped from 30.0 (1992) to 16.5 (2010) and Australia dropped from 34.3 (1994) to 23.5 (2011), an almost a 50 % relative reduction in the USA compared with only 25 % in Australia. Mortality rates in the USA and Canada have shown a greater decline since the mid-1990s than Australia, whereas England reached a similar rate to Australia in the early 2000s and has stabilised at a slower and less consistent rate.

Importantly, these results show that, had Australia experienced mortality rates similar to those of the USA, almost 11,000 deaths from prostate cancer might have been averted (1994–2010). Australian cancer patients have the highest estimated survival in the world (30 deaths for every 100 new cases for all cancers [13]. However, for prostate cancer, there are 14 deaths for every 100 new cases in Australian men, whereas in the USA, there are 11 deaths for every 100 new cases [13]. When compared to other common cancers, we can see that there are fewer deaths in Australians with lung and colorectal cancer (69/100 and 24/100, respectively) than in Americans (74/100 and 37/100, respectively). However, for breast cancer (female only), there is no difference between the two countries with 16 deaths per 100) [13]. This suggests that cancer control measures in Australia generally result in better outcomes than in the USA, with the exception of prostate cancer, increasing the imperative to better understand prostate cancer trends. The disparities between these countries are not well understood, and our analysis highlighted time periods when mortality changed significantly.

### PSA testing and prostate cancer incidence

Prostate cancer incidence rates are largely influenced by PSA testing. Key randomised controlled trials identified small mortality benefits associated directly with PSA testing, but this has not resulted in global population-wide screening recommendations to date as the evidence is inconsistent [3, 14–17]. Generally, men over 50, or over 40 with a family history of prostate cancer, are encouraged to discuss the possibility of PSA testing with their doctor, but updated US guidelines now recommend against PSA testing [18].

Differences in the uptake and use of PSA testing in each of the countries in this study are likely to account for a major proportion of incidence rate differences [12]. Over the time period analysed, PSA testing recommendations from peak bodies in each country varied. While no formal screening programs existed, PSA testing was generally widespread in certain areas with men encouraged to make informed decisions [19]. The current guidelines in the USA, UK and Canada do not recommend PSA testing for healthy, or asymptomatic, men [18, 20–22]. Australia’s recently released updated draft guidelines support testing every 2 years for healthy men between 50 and 69 if requested, and after sufficient information is provided on the benefits and harms, but PSA testing is not supported in individuals with <7 years’ life expectancy [23]. Variations to national recommendations similar to the Australian approach are also seen in some regions of Canada [24].

However, a direct comparison of testing rates in each country is difficult due to the lack of centrally held relevant data. Since the introduction of PSA testing in 1988, Australia has demonstrated higher rates of negative transrectal ultrasound-guided systemic biopsies and a higher incidence of low/intermediate grade prostate cancer in males younger than 55 in which cases would have gone previously undetected [25]. This may have contributed to the rise in incidence of Australian men under 65 from 1999 to 2008 (APC: 10.3 %).

Incidence rates in the USA and Australia decreased in the most recent period, despite the high rates of PSA testing reported with 75 % of men over 50 tested in the USA [26] and 64 % of men between 40 and 74 having been tested at least once in Australia [27]. The emerging evidence of a reduction in incidence in the USA and Australia is not immediately explicable, perhaps due to the more conservative use of PSA testing. Data from the USA support this possibility showing that PSA testing dropped after the updated guidelines [28, 29]. However, these studies also incorporate data predating this recommendation so future rates may decline further.

Conversely, the incidence of prostate cancer in England and Canada continued to increase during the most recent period of analysis. The lower UK incidence rates in comparison with the other countries are considered to be due to consistently low PSA testing rates. In 2007, the screening rate of asymptomatic men from 45 to 89 in the UK was 6.2 %, and prior to this, PSA testing was discouraged [8, 30]. In Canada, 53.8 % of males over 35 years of age had a PSA test in 2008 [31], with standard practice incorporating annual PSA testing [22].

### Prostate cancer treatment and mortality

While the changes in incidence are likely due to changes in testing behaviours identifying the causes for changes in prostate cancer mortality trends is more challenging. Early reports investigated the impact of PSA testing on prostate cancer mortality, and estimates suggest that the potential survival benefits take over 10 years to accrue [3, 32]. As a consequence, the drop in mortality, first apparent in Australia just 6 years after the introduction of PSA testing, was too soon to be a result of screening. If PSA testing were to have made a high impact on mortality, an accelerated rate of decreasing mortality would have been evident earlier. A similar conclusion was drawn from a Scottish study where increased detection did not clearly drive decreasing prostate cancer mortality [33].

The recording and coding of cause of death have been excluded as a driver of prostate cancer mortality changes with good agreement found between cause of death reported on death certificates and medical records in prostate cancer patients in the USA [34, 35]. However, a UK study suggested that changes to national coding practices resulted in an artificial increase in prostate cancer mortality from 1984 to 1992 and a decline after 1993 [36]. However, our joinpoint analysis for Australia, England and Canada showed little evidence of any increase in mortality during the first years of PSA testing, meaning misattribution was unlikely to be a major cause of any rise prior to the sustained fall observed.

The reduction in mortality in all four countries is likely due in part to improvements in treatment which is associated with improved survival [35, 37], especially of men with localised disease. Much of the prostate cancer treatment innovation has historically been initiated in the USA [38]. For example, radical prostatectomy (RP), for the treatment of localised prostate cancer, has higher reported usage in the USA. Its effectiveness in reducing prostate cancer mortality and the risk of disease progression was shown in men under 65 with 23 years follow-up [39], but in men under 75 years of age, the reduction in mortality was not statistically significant when compared to watchful waiting over a 10-year period [40]. The theory that diverse treatment methods are a major driver of disparities in mortality has not been conclusively proven and requires further investigation [38, 41]. RP treatments are now the most frequent treatment method used among men with clinically localised prostate cancer in Australia with decreasing use of hormonal therapy as a primary treatment [42]. From 2000 to 2006, there was a 53 % increase in RPs in England resulting from an increasing number of surgeons with the ability and willingness to perform the procedure [43]. However, a comparison of prostate cancer mortality in the USA and England showed significantly lower mortality rates in the USA, possibly explained by the lower prevalence of radical therapy in England [44]. While direct comparisons are not simple, evidence of differences in international variations in the use of active surveillance, local treatment and systemic therapy has been documented during the period of study [45].

Once clinical innovations are demonstrated, there is often a delay in their integration into routine practice. For prostate cancer treatment, the diffusion of robotic RPs was greater in the USA. By the mid-2000s, over 5,000 robotic RPs were performed worldwide of which approximately 4,800 were in the USA [46]. On the other hand, in Australia, robotic surgeries increased from 3 in 2003 to 2,775 in 2011, with robotic RP being the most common [47]. The impact of robotic RP on mortality is still being investigated as their effectiveness has been largely identified in the improvement in functional, patient-related outcomes rather than survival outcomes but this requires further investigation [48, 49]. However, these findings illustrate the higher and more immediate use of innovative techniques in the USA compared with other countries. Future innovations, such as the use of multi-parametric magnetic resonance imaging in the diagnosis of prostate cancer, to confirm staging and grading as well as planning for treatment, may further improve outcomes [50].

Much of the current debate in the medical, public health and broader communities is the high diagnosis rate and overtreatment of prostate cancer, especially low-risk cases [3–5, 14, 15, 32, 39–43, 51]. It is possible that higher screening prevalence in the USA, which occurred earlier than in other countries, may have detected more later stage prostate cancer cases and could have led to subsequent curative treatment or prolonging life sufficiently that the cause of death was not attributed to prostate cancer. Again, the comparison of national data collections on disease stage is hampered by the lack of internationally comparable data. The limited information available on prostate cancer treatments in Australia has been recognised, and, as a result, initiatives are also underway to compile a National Prostate Cancer Registry, based on the successful Victorian model [52]. However, a Swedish study suggests that the intensity of opportunistic screening has a greater effect on reducing prostate cancer mortality [53]. This may have contributed to the lower mortality in the USA, but the high levels of opportunistic screening in Australia should have results in similar mortality reductions, which are not evident in the available data.

A lack of systematic follow-up and treatment for men with high-risk disease after initial treatment may also be a potential area for improvement. Data from the USA suggest a more aggressive approach in these patients with high-risk features may be associated with differences in death rates. The use of ADT alone has been shown to reduce death from prostate cancer but may increase the risk of death from competing causes [5]. Recent evidence from randomised controlled trials has shown that overall survival of patients with locally advanced prostate cancer increases if they are given radiotherapy in addition to ADT, rather than the traditional treatment of ADT alone [54, 55]. Chemotherapy is considered an effective treatment for later stage prostate cancer, but it has lower uptake in older men, requires further lines of treatment to reduce the risk of death [6, 56] and likely only contributes a very small proportion of years of life gained in men treated.

The differences in healthcare systems in all four countries are likely to be partially responsible for differences in mortality trends [57] However, a previous study compared the divergence between USA and UK prostate cancer mortality trends with all-cancer mortality, finding little difference between the two [38] The study suggested that the detection or treatment differences for prostate cancer were likely the cause of differences between the two countries rather than any difference in systemic healthcare approaches [38].

### Limitations

The main limitation of our study is the ecological approach taken. This is an observational study using routinely available population-wide statistics. While it allows us to quantify the difference between Australia and other similar developed countries, it restricts the detailed interpretation of results and our ability to identify the reasons for the observed differences, and thus, it has reduced clinical relevance. An understanding of broad population-wide approaches to prostate cancer suggests that diagnosis and treatment patterns contribute to the differences. The possible effect of genetic or lifestyle factors on incidence and mortality has not been considered and is a limitation of this study. Existing publicly available data sources are not currently sufficient to explore the possible relationships on a population-wide level that have been suggested in previous work [58].

## Conclusions

In Australia, it is projected that there will be 25,000 new cases of prostate cancer by 2020 [59], contributing an estimated large component of healthcare expenditure relating to male cancers. While the declining mortality is clearly evidence of progress being made in controlling prostate cancer, our understanding of the reasons for this improvement is limited. Overall, the change in mortality appeared too early to be solely attributed to PSA testing, it is more likely the results of advancement in treatment, both for men with localised disease but also and perhaps more importantly men with high-risk disease [30]. We have confirmed that Australian prostate cancer mortality is not declining at rates comparable with other developed countries. Almost 11,000 deaths from prostate cancer may have been averted between 1994 and 2010 if Australia’s prostate cancer mortality rate had been equal to that of the USA. Further emphasis should be placed on confirming why this inequality in outcomes existed with more detailed information such as stage at diagnosis, treatment methods, screening techniques and diagnostic methods that may have influenced mortality trends. Continued effort and emphasis should be placed on monitoring patterns of care and outcomes of prostate cancer patients, and, as appropriate, system-wide differences should be addressed to reduce the number of men dying from this disease.
